# Analysis of Epidemiological Changes and Prevention Effects for Malaria in Weifang, Shandong Province, China from 1957 to 2017

**DOI:** 10.18502/ijph.v49i10.4687

**Published:** 2020-10

**Authors:** Xiao SONG, Qiqi SHI, Chongxing ZHANG, Xiangli KONG, Yeyuan LV, Haifang WANG, Hongmei LIU, Lijuan LIU, Xiuxia GUO, Jingxuan KOU, Xiaodan HUANG, Huaiwei WANG, Peng CHENG, Maoqing GONG

**Affiliations:** 1. Shandong Institute of Parasitic Diseases, Shandong Academy of Medical Sciences, Jining 272033, Shandong, China; 2. School of Medicine and Life Sciences, University of Jinan-Shandong Academy of Medical Sciences, Zhangqiu 250200, Shandong, China

**Keywords:** Malaria, Epidemiology, Prevention measures, Elimination, Weifang

## Abstract

**Background:**

We aimed to conduct a retrospective analysis of the epidemiological changes and prevention effects for malaria in Weifang, Shandong Peninsula, China from 1957 to 2017.

**Methods:**

The malaria data from a web-based reporting system were analyzed to explore malaria epidemiological characteristics and prevention effects in Weifang.

**Results:**

Overall, 1, 704, 890 malaria cases were reported in Weifang from 1957 to 2017, of which two major malaria epidemics occurred in 1961 (827.28/10, 000) and 1971 (366.14/10, 000). Prior to 1997, all malaria patients (1, 704, 829) were infected with *Plasmodium vivax* (*P. vivax*). After 2007, the cases of *Plasmodium falciparum* (*P. falciparum*) showed an upward trend (76.8%). The reported cases after the 21st century were mainly imported cases, and the last indigenous case was a patient that infected with *P. vivax* in 2006. Overall, 36 imported cases were reported from 2010 to 2017, of which 88.9% were acquired in Africa. Except for one 32-year-old woman, the rest were male (97.2%), in which laborers and farmers represented the vast majority (66.6%). From 1987 to 2017, there were 1, 224, 474 cases of fever with blood tests, and the average blood test rate was 4.9%. From 1957 to 2017, a total of 1, 704, 890 malaria patients were treated, 96 cases were treated during resting phase from 1987 to 2017.

**Conclusion:**

Weifang should continue to strengthen the management of the migrant population, making blood tests for fever patients and patient treatment as important means of malaria control and monitoring.

## Introduction

Malaria is a highly sensitive insect-borne infectious disease that is widely distributed in tropical, subtropical and temperate regions (1, 2). Humans malaria is caused by intraerythrocytic protozoa of the genus *Plasmodium* (mainly including: *P. vivax*, *P. falciparum*, *P. ovale* and *P. malariae*), all of which transmit the disease through the bite of female *Anopheles* mosquitoes (3). Updated estimates have suggested that 216 million malaria cases occurred globally in 2016, among which 445, 000 had fatal outcomes (4). In China, *P. vivax* and *P. falciparum* have been prevalent for many years and have a high incidence historically (5). After decades of large-scale national continuous efforts (e.g., effective vector control, active case detection strengthening of health systems, improving case management, and enhanced case reporting and surveillance) (6), the annual incidence of malaria in China has dropped from more than 20 million in the early 1970s to <10, 000 by the middle of 2010s (7, 8), only 3116 cases of malaria were reported in 2015 (8). Furthermore, China initiated the National Malaria Elimination Action Plan (NMEAP) in 2010, which aimed to eliminate indigenous malaria in 2020 (9). Shandong province used to be a high malaria transmission area in China (10), the malaria annual morbidity has reached two peaks in 1961 (806.2/10, 000) and 1971 (493.2/10, 000), respectively (11). Since then, the disease was significantly controlled due to large-scale investigations and anti-malaria campaigns in areas with high malaria transmission.

Weifang, Shandong Province, had been once a high *P.vivax* malaria endemic area with *Anopheles sinensis* as the single vector. After decades of active prevention and control work, there are no more indigenous cases. Nevertheless, in recent years, imported malaria has become an increasingly serious problem in Weifang, and the changing characteristics of these malaria cases is rarely known. This article summarizes the epidemiological characteristics of malaria in Weifang from 1957 to 2017, as well as the active measures taken for prevention and control of malaria, with the aim to provide scientific insights for further development of targeted malaria control strategies and to accelerate the goal of elimination of malaria in Shandong Province.

## Methods

### Study setting

**Fig. 1:**
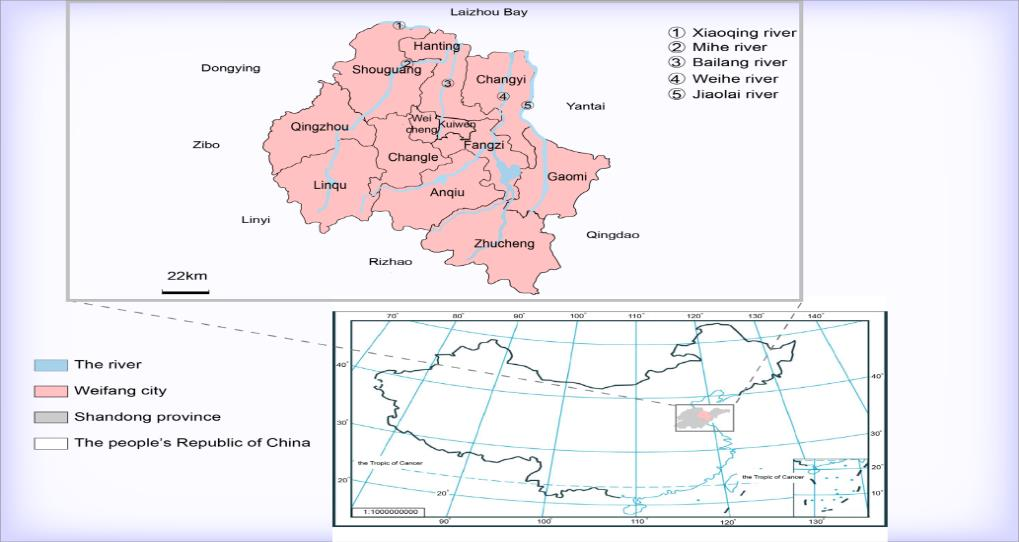
Location of Weifang in Shandong province of China. The names of 12 counties (cities) are labelled above, and the five major river systems associated with *An.sinensis*breeding are marked in blue

Weifang lies in the central part of the Shandong Peninsula, adjacent to Linyi, Rizhao, Qingdao and Yantai between 35°41′-37°26′ north latitude and 118°10′-120°01′ east longitude. It administers 4 districts, 6 cities and 2 counties, with 5 urban development zones currently (Fig. 1). According to the website of the local meteorological department, Weifang’s annual average temperature and the precipitation are 12.9 °C and 605.8 mm, respectively. Weifang is located in the north temperate monsoon region, with a warm temperate monsoon type, semihumid continental climate. There are 103 rivers with a drainage area of more than 50 km^2^ and five major river systems including the Weihe River, the Mihe River, the Bailang River, the Jiaolai River and the Xiaoqing River (Fig. 1). The rural economy primarily depends on agriculture and labor export.

### Data extraction

The data were obtained from the web-based reporting system (WBRS) and compilation of information on the elimination of malaria, (i.e., epidemic situation report forms, individual case information report forms and summary of prevention and control efforts).

### Cases types

Indigenous malaria was defined as any cases that infected in the province with no history of travel or unfalsifiable locally acquired transmission (12). Imported cases were defined as patients with malaria infections traced to origins in a malaria-endemic country within the previous month or those traveled to another district within China, but diagnosed in a nonendemic area after development of the clinical disease (13, 14).

### Fever patient blood smear microscopy

Since 1975, Weifang has carried out microscopic examinations on “four fever” patients (clinically diagnosed as malaria, suspected malaria, cold and fever, and unexplained fever) from May to Oct each year. Since 2010, the city has considered “triple fever” patients (clinically diagnosed as having malaria, suspected malaria or unexplained fever) and conducted blood tests for malaria parasites.

### Analysis and statistics

Statistical analysis was conducted by using Microsoft Office Excel 2007 (Microsoft Corp., Redding, WA, USA) and SPSS software (Version 19.0 for Microsoft Windows, SPSS Inc., Chicago, USA).

## Result

### Overall epidemiologic profile

From 1957 to 2017, 1, 704, 890 malaria cases were identified in Weifang, with an incidence of 1, 766.93/10, 000. During 1960–1979, there had been a high incidence of malaria, with 1, 693, 203 malaria cases, including two major malaria outbreaks in 1961 and 1971, with incidences of 827.28/10, 000 and 366.14/10, 000, respectively. The interval 1980–1988 was the period of basic malaria control, with 6, 019 malaria cases reported in the city. The highest incidence was in 1980 (3, 042 cases) with an incidence of 4.02/10, 000. Subsequently, the incidence of malaria declined annually. In 1988, the incidence of malaria was only 0.01/100, 000. After the assessment of the Provincial Health Department, elimination of malaria was achieved. From 1989 to 2009, 36 cases of malaria were reported in the early stages of malaria elimination. The highest incidence rate was 1998 (0.06/100, 000), and the average incidence of malaria was 0.02/100, 000 in the remaining years; in 1989, 1996, 2001, and 2002, no malaria cases were reported. The last indigenous case was a case of *P.vivax* reported in Fangzi district in 2006, after which no local cases occurred for 11 consecutive years. In the elimination process from 2010 to 2017, a total of 36 imported cases of malaria were reported, with 1 patient died of *P. falciparum* (Fig. 2a, b).

**Fig. 2a:**
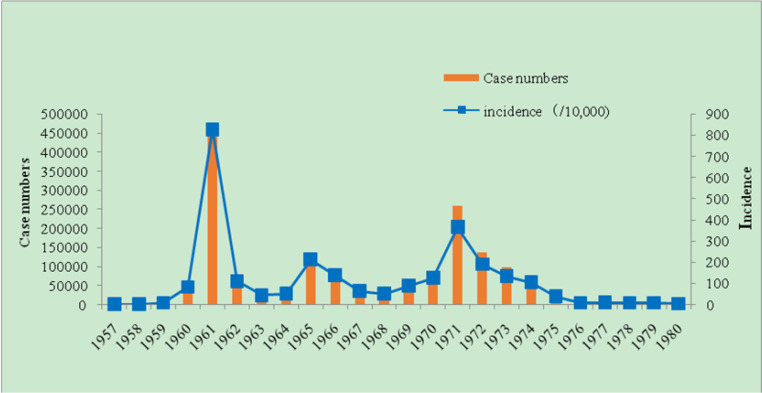
Malaria case numbers and incidence reported during the period in Weifang, Shangdong Province from 1957–1980

**Fig. 2b:**
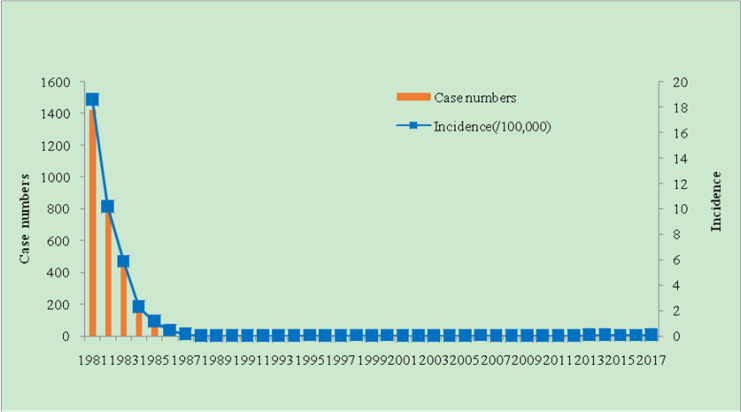
Malaria case numbers and incidence reported during the period in Weifang, Shangdong Province from 1981–2017

### Plasmodium species composition

From 1957 to 2017, three species of *plasmodium* (*P. vivax*, *P. falciparum* and *P. ovale*) were identified in 1, 704, 890 malaria cases. Prior to 1998, all cases of malaria were caused by *P. plasmodium* infection and included 1, 704, 829 cases. Since that year, the cases of *P. falciparum* and unclassified d *Plasmodium* infection have been reported. From 2007 to 2017, 33 cases of *P.falciparum* were reported, accounting for 76.8% of the total number. In addition, two cases of *P.ovale* infection were identified in 2013 and 2017. Furthermore, there were some unclassified *plasmodium* infections, accounting for only 2.3% of total malaria infections in 2007–2017 (Table 1).

**Table 1:** *Plasmodium* species in Weifang from 1957 to 2017

***Year***	***P. vivax***	***P. falciparum***	***P. ovale***	***Mixed***	***unclassified***	***Total***
1957–1966	852, 687	0	0	0	0	852, 687
1967–1976	827, 289	0	0	0	0	827, 289
1977–1986	24, 828	0	0	0	0	24, 828
1987–1996	23	0	0	0	0	23
1997–2006	10	6	0	0	4	20
2007–2017	7	33	2	0	1	43
Total	1, 704, 844	39	2	0	5	1, 704, 890

### Geographic distribution of imported cases

**Fig. 3:**
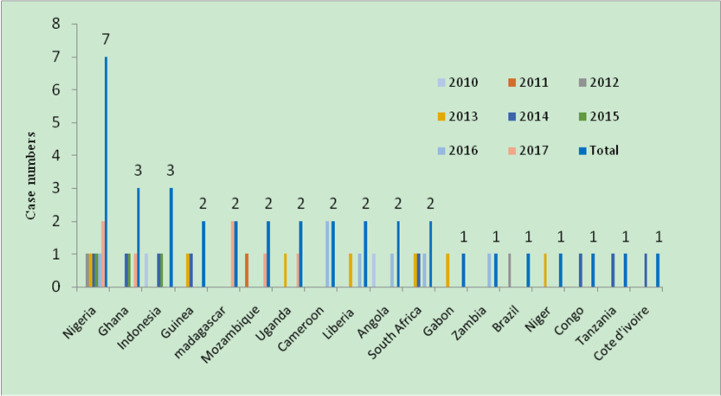
Source countries of imported malaria from Africa, Southeast Asia and Latin America, 2010–2017, Horizontal line shows the source countries of imported cases and vertical line shows case numbers

From 2010 to 2017, all cases were imported from other countries. Among the cases imported from abroad, 88.9% were in patients returned from African countries. A proportion of 8.3% came from Southeast Asia (Indonesia), and 2.8% were derived from Latin America (Brazil). In 32 African cases of imported malaria, 53.1% were derived from West African countries: 7 patients were infected in Nigeria; 3 in Ghana; 6 patients in Liberia, Guinea and Angola; and one in Cote d’ivoire. A proportion of 21.9% cases were derived from Eastern Africa countries with 2 patients each infected in Uganda, Madagascar and Mozambique, and one patient infected in Tanzania. A proportion of 18.8% came from Central African countries: 2 patients were infected in Cameroon, and 1 patient each was infected in Gabon, Zambia, Congo and Niger. A proportion of 6.2% were derived from South African countries, but the countries were unidentified (Fig. 3).

### Demographic features of imported cases

**Fig. 4:**
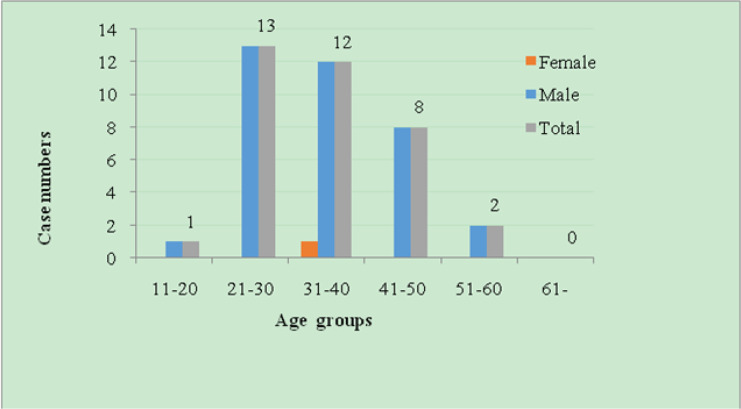
Age and Sex distribution of imported malaria cases reported in Weifang, Shangdong Province from 2010–2017

**Fig. 5:**
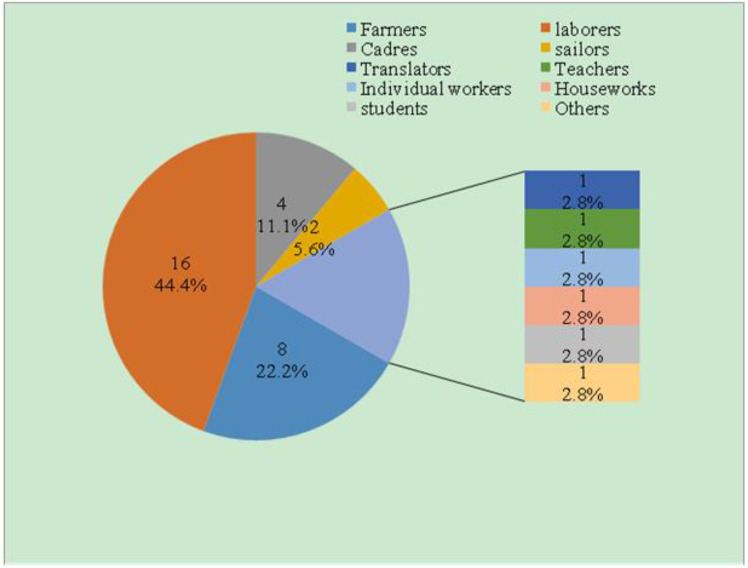
Occupation distribution of imported malaria cases, 2010–2017. Different colors in the pie graph represent differenct occupations

The age distribution of malaria cases in 2010–2017 is shown in Fig. 4. The 21–30 age group had the most malaria cases, accounting for 36.1% of the total cases, followed by the 31–40 and 41–50 age groups, accounting for 33.3% and 22.2% of the total cases, respectively. The remaining 11 to 20 and 51 to 60 age groups included only a few cases, accounting for 2.8% and 5.6% of the total malaria cases, respectively. Among the 36 imported cases, there was only one 32-year-old woman, and the rest were males, comprising 97.2% of the total cases. Among them, laborers and farmers were the most represented, accounting for 44.4% and 22.2% of the total occupational distribution, respectively; 4 cadres accounted for 11.1% of the total cases; and 2 sailors accounted for a relatively lower risk (5.6%) compared to the cadres. The remaining occupations included students, translators, teachers, sole proprietors, houseworkers and others who accounted for approximately 2.8% of the total cases (Fig. 5).

### Temporal distribution of imported cases

**Fig. 6:**
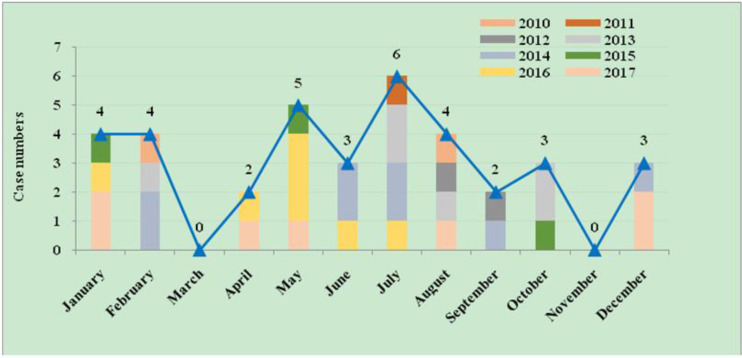
Monthly distributions of imported malaria cases, 2010–2017. Horizontal line shows 12 monthly and vertical line shows case numbers. The Stacked-coloum of different colors and the fold line show change trend of the imported numbers monthly from 2010–2017

According to the Fig. 6, the time of onset of imported cases was primarily from Jan to Feb at the beginning of the year and from May to Aug after entering the summer, accounting for 72.2% of the total number of infections. Among them, July accounted for 16.7% of the total cases, reaching the peak of infection. After Aug, the incidence gradually declined, and no cases were reported in November and March.

### Malaria prevention and control work

#### Fever patient blood tests

From 1987 to 2017, a total of 1, 224, 474 cases of fever were detected in blood, and the average blood test rate was 4.5%. Among these, there were 888, 506 fever patients with blood tests, and the average annual blood test rate was the highest (15.4%) during 1987–1993. The number of fever patients with blood tests decreased from 1994 to 2001, and the average blood test rate was 2.9%. In 2002–2009, the average blood test rate was 0.4%, according to relevant program requirements. The number of blood fever patients in 2010–2017 was higher than that in 2002–2009, and the average blood test rate was 1.5% (Table 2).

**Table 2:** The number of blood tests, patients treated and preventive medication in Weifang from 1987 to 2017

***Year***	***Blood tests***	***Mean blood tests rate (%)***	***Cases treated***	***Patients treated of resting phase***	***Preventive medicine***
1987–1993	888506	1.54	5	71	115629
1994–2001	193919	0.29	16	11	32955
2002–2009	29183	0.04	16	5	0
2010–2017	112865	0.15	29	10	0
Total	1224473	2.02	73	97	148584

### Malaria patients’ treatment and group prevention medication

From 1957 to 2017, 1, 704, 890 malaria patients were treated in Weifang, of which, 1, 704, 818 malaria patients were treated during 1957 to 1988. After basic eradication of malaria, 36 cases of malaria were treated from 1989 to 2009. Since the launch of the campaign to eliminate malaria in 2010, Weifang has been actively managing currently sick patients. Forty-three cases of malaria were treated, and all patients were in good condition after treatment, except for one who died. From 1987 to 2017, 97 patients were treated during the resting phase; 87 of the patients were treated during the resting phase from 1987 to 2009, and nine patients with *P. vivax* and *P. ovale* have been treated during the resting phase since 2010. From 1960 to 1997, approximately 21.5 million people took preventive medication, among which 148, 584 patients took preventive medication from 1987 to 1997. After 1997, according to the requirements of the program, Weifang only conducted a standardized resting phase for the treatment of vivax malaria and no longer carried out group preventive medication work (Table 2).

### Antimosquito campaign and mosquito-borne monitoring

From 1964 to 1978, more than 6 million breeding sites were treated in the whole region, effectively controlling the source of infection and the media of transmission. During the breeding season of mosquito vectors from 2000 to 2005, 8, 976 mosquitoes were caught in human nets, of which 92 were *An. sinensis*, accounting for 1.02% of the total plasmodia. The average densities of *Anopheles* mosquitoes from July to Sep were 0.12, 0.41 and 0.36/account. There were 4253 mosquitoes in the cattle sheds, of which 42 were *An. sinensis*, accounting for 0.98%, and the average densities of *Anopheles* were 0.25, 0.57 and 0.33/account from Jul to Sep. From 2012 to 2016, 312 mosquitoes were caught in Zhucheng county by the mosquito trap lamp method, and no *An.sinensis* were caught. In 2016, 70 mosquitoes were caught by using the human trap method, and there were also no *An.sinensis*.

### Business training

From 2011 to 2016, Weifang trained 3430 malaria microscopic inspection personnel and epidemic management personnel. Of which, 1311 microscopic inspection personnel, 427 epidemiological personnel and 204 media investigators were trained from 2011 to 2012. In 2013, more than 40 chief of infectious diseases section and epidemic management personnel were trained, and from 2014 to 2016, there were 1448 person-times of malaria microscopy training.

## Discussion

Weifang used to be a high *P. vivax* malaria endemic area with *An. sinensis* as a single vector, and there are five major river systems as well as numerous branch rivers which run through the whole city and towns of Weifang. However, these branch rivers with reeds, irrigation canals, ponds and drainage ditches in the village surroundings have been found to be the best breeding sites for *An. sinensis* (15). While, residents who live nearby are vulnerable to be bitten by mosquitoes at peak biting times and thus infected with malaria.

In the late 1950s, the river systems was flooded due to continuous and intense heavy rains and was not received effective comprehensive control. The mosquito larvae would multiply rapidly in the temperature-increasing season after the water source was polluted, the first malaria pandemic was formed in 1961 and brought a disaster to the local people (16). Since the first malaria outbreak, the city has organized a strong anti-malaria work team to reduce malaria transmission. Therefore, after 1962, the incidence of malaria has declined annually. Unfortunately, after the “Cultural Revolution” began in 1966, the order was chaotic, most of the anti-malaria teams were disbanded, and malaria prevention and control measures could not be well implemented. As a result, the incidence of malaria rose again in 1971, forming the second peak. Thus, the whole city actively carried out prevention and treatment work after the outbreak of malaria, combined with a mass patriotic health campaign to eliminate mosquito breeding places, to perform timely treatment of current patients, and to implement national preventive medication work, strict training of health personnel at all levels forms an extremely complete anti-malaria network. Due to proper measures, the incidence of malaria has dropped significantly from 1972 to 1979. Since 1980, the whole city has improved its monitoring system, requiring all medical and health units to be equipped for microscopic examination to make blood tests for malaria parasites a routine practice to effectively control the epidemic. Until 1988, the basic malaria elimination standard of the Ministry of Health had been achieved. From 1989 to 2009, the incidence of malaria in the city showed a steady declining trend; many malaria-free counties emerged, and the focus of future work shifted to implementation of purification measures after basically eliminating malaria, emphasizing the management of migrant population and key populations to search for sources of infection. Due to effective measures, the incidence of malaria has stabilized at a relatively low level over more than ten years, effectively consolidating the results of prevention and control.

During the 21st century, as international trade and the number of people going abroad increased, especially the number of migrant workers in Africa and Southeast Asia, imported cases gradually replaced indigenous cases. The age, gender and occupation distributions indicate that males of 21–40 yr old were at a high risk for imported malaria infection, the main occupations of imported malaria patients were laborers and farmers, since they frequently engage in activities such as mining, logging, farming and construction in Africa and Southeast Asian countries that are severe malaria-endemic areas and they are vulnerable to be bitten by female *Anopheles* mosquitoes at peak biting times that causes disease and even death (17–19). The analysis in this study suggested that the seasonal fluctuation of imported malaria cases was relatively weak, with peaks occurring in Jan to Feb and May to Aug. Chinese New Year holidays are celebrated in Jan to Feb, and laborers and farmers return to China from malaria-endemic countries to engage in agricultural work during May to Aug (20). Interventions strengthening their awareness of the risks of malaria and occupation-based vector control measures should be implemented to reduce the risk of malaria infection among export laborers.

In this study, indigenous cases were caused by infection with *P. vivax*, carried by *An.sinensis*, and most of the imported cases originating in Africa were caused by *P.falciparum*. It is the most pathogenic malaria species, with the highest incidence in sub-Saharan Africa. Since *P. vivax* can survive at lower temperatures and higher altitudes, it has a broader geographic range than *P. falciparum. P. vivax* and *P. ovale* commonly lead to malaria recurrence, which has a dormant liver stage (hypnozoites) that can reactivate months or years after the acute infection. Therefore, clinicians and public health workers should strengthen the follow-up treatment of *P. vivax* and *P. ovale* (13). Due to its morphological similarities and tertian periodicity, *P. ovale* can easily be mistaken for *P. vivax* or *P. malariae* on microscopic examination (21, 22). However, misdiagnosis can lead to the overtreatment with medications, increasing the risk of disease and even death. Therefore, ensuring the accurate diagnosis will facilitate early detection and effective standard treatment for malaria cases. There are usually three methods to diagnose malaria: microscopic examination of Giemsa-stained thick and thin blood smears, RDTs and PCR (23, 24). In general, microscopic examination was considered the gold standard for immediate diagnosis (25).

Since the NMEAP was launched in 2010, the prevention and control of malaria in Weifang has mainly started with blood tests for fever patients, standardized treatment of epidemics and health education. By Dec 2015, 12 counties and cities have passed the county-level assessment of malaria elimination in Shandong Province. China is one of the member countries of APMEN who successfully established a good surveillance and response system known as the “1-3-7” strategy (case notification within 1 day, case confirmation and epidemiological investigation within 3 d and focused investigation and response to prevent further transmission within 7 d of malaria diagnosis) (26–29). Weifang strictly implements this malaria epidemic management mode to actively manage the imported cases. In addition, primaquine is used to treat malaria patients before June every year for antirecurrence treatment. Temporary household registration and blood collection examinations were carried out for the nonlocal population from the area of high malaria, and for those with positive blood tests or a history of malaria, and radical treatment was given according to the infected species. At present, most malaria patients have a better prognosis and no recurrence occurs.

However, there was one vivax malaria patient from Ghana who died from multiple organ failure due to the critical condition. This undoubtedly sounded the alarm for us. Although Weifang has achieved certain success in the eradication of malaria, there are still some problems and weaknesses in the work. Therefore, in future work, effective management of surveillance and response system should be strengthened and improved to ensure timely detection and prompt response to individual cases efficiently (30), especially among laborers and farmers after returning from severe malaria-endemic areas (31).

## Conclusion

Although the incidence of the indigenous malaria in China has dropped to very low levels, increasing imported malaria cases from Africa and Southeast Asia remain a major threat. Therefore, it is necessary to eliminate potential reservoirs and prevent malaria outbreaks caused by imported pathogens. At the same time, attention should be given to strengthening capacity in monitoring, emergency response, diagnosis and treatment.

## Ethical considerations

Ethical issues (Including plagiarism, informed consent, misconduct, data fabrication and/or falsification, double publication and/or submission, redundancy, etc.) have been completely observed by the authors.
